# Regional citrate anticoagulation for renal replacement therapy during venovenous ECMO: A randomized crossover pilot study

**DOI:** 10.1016/j.aicoj.2026.100072

**Published:** 2026-04-27

**Authors:** Marco Giani, Marta Frazzei, Roberto Rona, Thomas Langer, Matteo Pozzi, Giuseppe Foti, Emanuele Rezoagli, the CRRT ECMO Study Group

**Affiliations:** a Department of Medicine and Surgery, University of Milano-Bicocca, Monza, Lombardy, Italy; b Emergency and Intensive Care, Fondazione IRCCS San Gerardo dei Tintori, Monza, Lombardy, Italy; c Emergency and Intensive Care Medicine, ASST Grande Ospedale Metropolitano Niguarda, Milano, Lombardy, Italy

**Keywords:** CRRT, ECMO, Regional citrate anticoagulation, Acute kidney injury

## Abstract

**Background:**

Regional citrate anticoagulation (RCA) is suggested as the preferred anticoagulation strategy during continuous renal replacement therapy (CRRT), as it prolongs circuit lifespan while minimizing bleeding complications. However, evidence on its use in CRRT circuits during extracorporeal membrane oxygenation (ECMO) is limited. In patients receiving ECMO, systemic anticoagulation with unfractionated heparin (UFH) is routinely administered to maintain circuit patency and is often relied upon to anticoagulate the CRRT circuit, limiting the use of regional citrate anticoagulation. The aim of this study is to evaluate whether adding regional citrate anticoagulation (RCA) to systemic unfractionated heparin (UFH) reduces CRRT circuits clotting in patients undergoing venovenous extracorporeal membrane oxygenation (VV ECMO).

**Results:**

Patients were randomized to receive alternating CRRT circuits anticoagulated with either systemic UFH alone or regional citrate anticoagulation added to systemic UFH (RCA + UFH), according to a predefined crossover sequence. Each circuit was maintained for up to 72 h or until clotting occurred. Coagulation parameters, CRRT pressures, and thromboelastography (TEG) data were collected.

A total of 66 CRRT circuits from 18 patients were analyzed (33 RCA + UFH; 33 UFH). No clotting events occurred in the RCA + UFH circuits, whereas 6 events were observed with UFH alone (0% vs 19%; *p* < 0.001). Cox proportional hazards analysis showed significantly longer circuit survival with RCA + UFH compared to UFH alone (p = 0.029). Platelet counts increased during RCA + UFH but declined during UFH alone (median change +6 vs −7 ×10³/μL; *p* < 0.001), with a significantly more favorable overall trend under RCA + UFH (effect estimate +13 × 10³/μL, 95% CI 8–19). D-dimer levels increased significantly during UFH alone, whereas a lower increase was observed with RCA + UFH (effect estimate −782 μg/L, 95% CI −1525 to −39).

Thromboelastography performed at the circuit level showed significantly prolonged R-times with RCA + UFH compared with UFH alone (median R-time 90 *vs.* 30 min; *p* < 0.001). No clinically relevant RCA-related metabolic complications were observed, including no episodes of severe hypernatremia, metabolic alkalosis, or citrate accumulation.

**Conclusions:**

In patients undergoing VV ECMO, adding regional citrate anticoagulation to systemic unfractionated heparin reduced CRRT circuit clotting, prevented platelet consumption. This technique was feasible, safe, and may improve CRRT efficiency in this high-risk population.

**Clinical trial:**

ClinicalTrials.gov Identifier NCT05148026

## Background

Extracorporeal membrane oxygenation (ECMO) is a rescue therapy for patients with severe acute respiratory distress syndrome (ARDS) [1]. In this population of critically ill patients, acute kidney injury (AKI) is a frequent complication [2], often necessitating continuous renal replacement therapy (CRRT) [3,4]. The use of extracorporeal circuits, such as those employed in ECMO and CRRT, requires effective anticoagulation to maintain circuit patency and prevent premature filter clotting, primarily driven by activation of the coagulation cascade following contact of blood with nonbiological surfaces [5,6].

Regional citrate anticoagulation (RCA) is suggested by the KDIGO 2012 international guidelines as the preferred anticoagulation strategy for CRRT, when not contraindicated, although the level of supporting evidence is limited [7]. This recommendation is supported by previous randomized controlled trials [8,9], which demonstrated that RCA, compared with systemic heparinization, significantly prolongs filter lifespan and reduces bleeding complications in critically ill patients with AKI. However, RCA cannot be applied to ECMO circuits, as the high blood flow rates required for extracorporeal gas exchange exceed the metabolic clearance capacity of citrate, which limits its application to extracorporeal systems operating at low blood flow rates, typically below 150 mL/min [10]. Consequently, systemic unfractionated heparin (UFH) remains the standard anticoagulant during ECMO support [11]. It has been suggested that, when CRRT is integrated into the ECMO circuit, systemic anticoagulation alone may be sufficient and that no additional circuit-specific anticoagulation is required [12]. However, this assumption remains controversial, as clotting within CRRT circuits frequently occurs in clinical practice [13], likely due to lower blood flow rates and the absence of heparin coating in CRRT tubing and filters. This may exacerbate platelet and fibrinogen consumption - a major concern in ECMO patients who are already prone to thrombocytopenia [14], coagulopathy, and bleeding complications [15]. Such events are associated with worse outcomes, prolonged ICU stays, increased transfusion requirements, and higher healthcare costs [16].

Previous studies have suggested that the use of RCA for CRRT integrated into an ECMO circuit is feasible and safe, and may reduce circuit clotting while improving filter patency [[17], [18], [19]]. In a previous retrospective study from our group [20], the use of RCA for the CRRT circuit in ECMO patients was associated with reduced circuit clotting without an increase in adverse events.

To validate these findings prospectively, we designed a randomized crossover pilot trial to determine whether the addition of RCA to systemic UFH, compared with UFH alone, reduces CRRT circuit clotting in patients supported with venovenous (VV) ECMO.

## Methods

### Trial design

This single-center, prospective, randomized crossover pilot study was conducted in the general intensive care unit (ICU) of IRCCS San Gerardo dei Tintori Hospital, Monza, Italy. The study was approved by the institutional ethics committee (October 2021; ref. 3732). According to Italian regulations, deferred informed consent was obtained from all patients once they regained consciousness.

The study design and all primary and secondary endpoints were predefined and prospectively registered on ClinicalTrials.gov (NCT05148026).

### Patient population

We included adult patients admitted to the ICU who required VV ECMO and concomitant CRRT for acute kidney injury (AKI) between November 2021 and July 2025.

Exclusion criteria were pregnancy; ECMO modes other than VV; contraindications to heparin or citrate; clinical conditions necessitating CRRT configurations different from the study protocol; and moribund status.

Contraindications to heparin or citrate included active bleeding, severe thrombocytopenia, heparin-induced thrombocytopenia, severe hepatic failure, refractory hypocalcemia, or high risk of citrate accumulation.

### CRRT and ECMO management

CRRT was performed using the MultiFiltrate system (Fresenius Medical Care, Bad Homburg, Germany). The CRRT circuit was integrated into the ECMO circuit through two dedicated side ports located across the membrane oxygenator. Specifically, the CRRT arterial (inlet) line was connected downstream of the oxygenator, whereas the venous (return) line was connected upstream, between the centrifugal pump and the oxygenator (see Fig. S1 – Supplementary material).

CRRT circuits were electively replaced every 72 h, following the manufacturer’s recommendations, or earlier if circuit clotting occurred. Circuit clotting was defined as visible thrombus formation within the circuit or clinically significant impairment of circuit performance requiring premature circuit replacement. This included progressive increases in circuit pressures (e.g., prefilter pressure and pressure drop or a transmembrane pressure > 300 mmHg) consistent with reduced filter patency [8].

Detailed information regarding cannulation strategy, anticoagulation protocol, CRRT and ECMO management is provided in the Supplementary Methods.

### Randomization and intervention

**Fig. 1:**
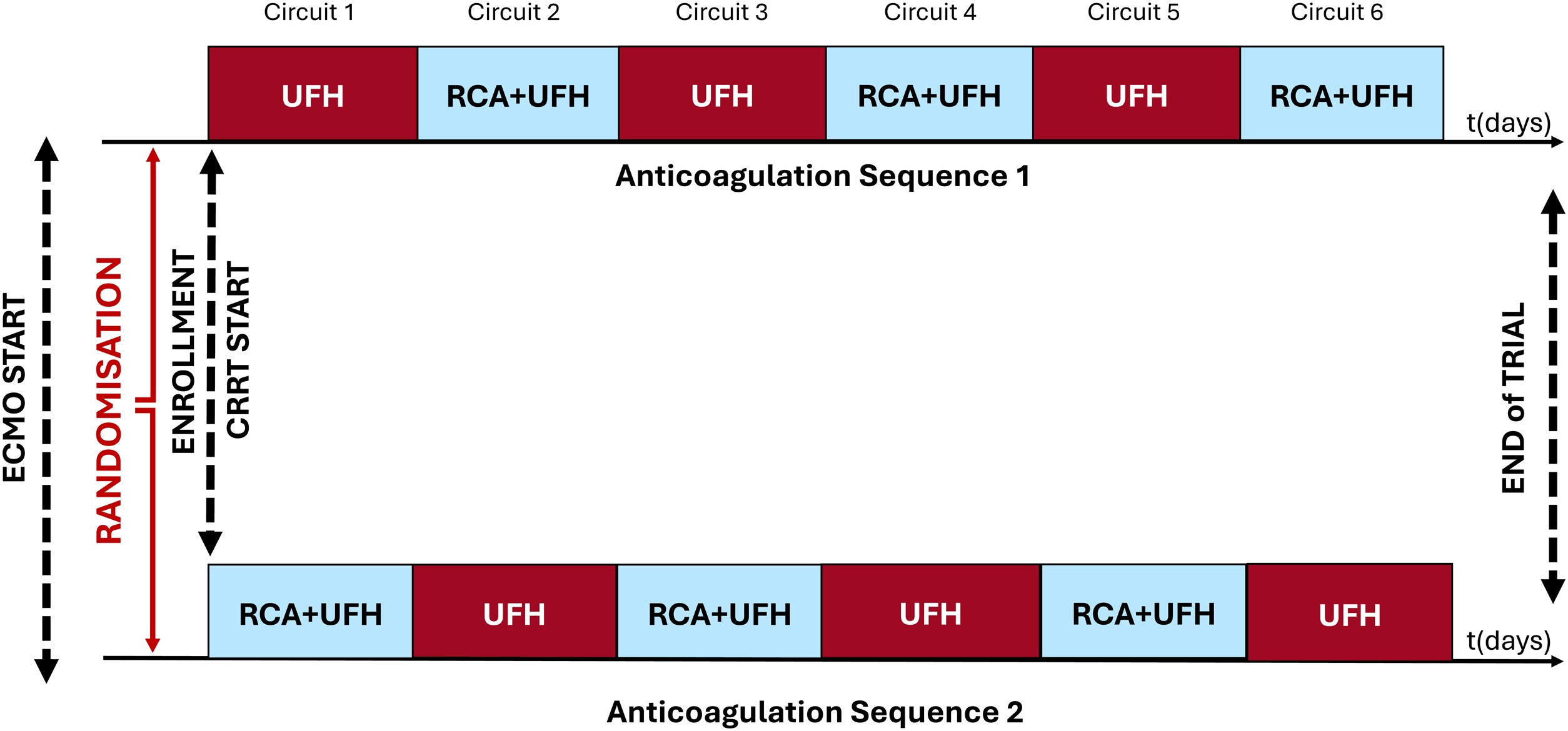
Study design and anticoagulation crossover sequences. Schematic representation of the randomized crossover study design. After enrollment and initiation of CRRT during VV ECMO, patients were randomized to one of two predefined anticoagulation sequences. In Sequence 1, CRRT circuits were anticoagulated with unfractionated heparin (UFH) alone followed by regional citrate anticoagulation plus UFH (RCA + UFH), alternating thereafter for up to six consecutive circuits. In Sequence 2, the order was reversed, starting with RCA + UFH and alternating with UFH alone. Each circuit lasted up to 72 h or until circuit clotting occurred. Systemic UFH anticoagulation for ECMO was maintained throughout the study period.

Patients were randomly assigned, using a computer-generated randomization sequence, to one of two anticoagulation sequences illustrated in Fig. 1.

In sequence 1, treatment began with UFH alone, followed by RCA + UFH, alternating for up to six circuits or until ECMO or CRRT discontinuation or death. In sequence 2, the order was reversed, starting with RCA + UFH and alternating accordingly.

This crossover design was chosen to minimize interindividual variability and allow each patient to serve as their own control, thereby reducing the potential influence of patient-specific factors on anticoagulation efficacy, coagulopathy, and adverse events.

### Endpoints

The primary endpoint was the incidence of CRRT circuit clotting according to the anticoagulation regimen.

Secondary endpoints included circuit survival, platelet count, D-dimer and fibrinogen levels, and the incidence of RCA-related complications. RCA-related complications were predefined as citrate accumulation (total-to-ionized calcium ratio > 2.5), moderate hypernatremia (plasma sodium 150–169 mmol/L) or severe hypernatremia (≥170 mmol/L), and metabolic alkalosis. Plasma sodium concentration was assessed using point-of-care blood gas analysis. (GEM® 5000 analyzer, Werfen, Barcelona, Spain). The anticoagulant effects of UFH, RCA, and their combination were assessed using thromboelastography (TEG), as described in the Supplementary Methods.

### Data collection

CRRT circuit pressures (prefilter, postfilter, and drainage pressures) and operating settings (blood flow, dialysate flow rate, and sodium citrate and calcium chloride infusion rates) were recorded every 2 h throughout the study period. The indexed filter pressure drop was calculated as the difference between prefilter and postfilter pressure, normalized per 100 mL/min of CRRT blood flow to account for differences in blood flow between patients and anticoagulation strategies. Full blood count (hemoglobin, hematocrit, white blood cells, and platelets), coagulation parameters (international normalized ratio [INR], activated partial thromboplastin time [aPTT], D-dimers, fibrinogen, and activated clotting time [ACT]), and arterial blood gas analyses were performed every 8 h.

Daily laboratory tests included liver and renal function, electrolytes, lactate dehydrogenase (LDH), C-reactive protein (CRP), procalcitonin (PCT), antithrombin III (ATIII), and anti-factor Xa activity (anti-Xa). Thromboelastography R-time was measured once per CRRT circuit, 2–6 h after CRRT initiation, using blood samples drawn directly from the CRRT circuit, to assess the anticoagulant effect of UFH, RCA, and their combination. Data on platelet pool transfusions and ATIII supplementation were collected at 24, 48, and 72 h after CRRT initiation.

### Statistical analysis

Continuous variables were expressed as median [interquartile range, IQR], and categorical variables as counts and percentages. Outcomes were analyzed at the circuit level, accounting for within-patient correlation inherent to the crossover design.

To account for the crossover design and the hierarchical structure of the data, mixed-effects models were used to compare the two CRRT anticoagulation strategies. The CRRT circuit was considered the unit of analysis. Patient ID was included as a random intercept to account for within-patient correlation due to repeated measurements across multiple circuits. CRRT anticoagulation strategy (RCA + UFH vs UFH alone), period (i.e., circuit order), and randomization sequence (i.e., initial treatment allocation) were included as fixed effects to appropriately model treatment, temporal, and allocation-related influences within the crossover framework. All p-values comparing variables measured during RCA + UFH versus UFH alone were derived from these models, including coagulation parameters (aPTT, anti-Xa activity, ACT), laboratory values, and circuit-related variables. Platelet count, fibrinogen, and D-dimer levels were analyzed both as absolute values and as relative changes from baseline, defined as the value at the start of each CRRT circuit.

Circuit survival, defined as the time from CRRT circuit initiation to circuit clotting, was primarily analyzed using a Cox proportional hazards model, with clotting as the event of interest. Circuits discontinued for reasons other than clotting (ECMO or CRRT discontinuation, or death) were right-censored. The model included anticoagulation strategy, period, and randomization sequence as covariates to account for the crossover design. Kaplan–Meier curves were additionally provided only for descriptive and graphical purposes. All tests were two-tailed, and a p-value < 0.05 was considered statistically significant. Statistical analyses were performed using JMP Pro 18.0 (SAS Institute, Cary, NC, USA).

### Sample size calculation

Based on previous data reporting a circuit clotting rate of 38% [20] with UFH alone and 11% with RCA + UFH, we estimated that 92 circuits (46 per group) would be required to detect a difference between treatments, assuming 80% power and a two-sided α = 0.05. Given that prior studies reported a median use of five CRRT circuits per patient, we planned to enroll 20 patients to achieve the target sample size.

## Results

The study included 66 CRRT circuits from 18 patients, with 33 circuits anticoagulated using RCA plus UFH and 33 using UFH alone. Enrollment was discontinued before reaching the planned 20 patients due to insufficient funding for study insurance. All enrolled patients completed at least two treatment periods according to the crossover protocol and contributed data for both anticoagulation strategies.

The median number of CRRT circuits per patient was 3.5 [2–5.25], with a median CRRT duration of 72 h [41.5–72], resulting in a cumulative CRRT exposure of 193 h [107–293] per patient. Cumulative CRRT exposure was comparable between groups (RCA + UFH: 103 [62.75–176.5] hours; UFH: 99 [70.5–144] hours).

Baseline clinical and demographic characteristics are summarized in Table 1.

**Table 1:** Baseline characteristics of the study population at randomization.

Variable	Value
**Baseline characteristics**	
Age, years	56 [48−63]
Females, *n* (%)	7 (39)
Body Mass Index, Kg/m^2^	28.9 [25.8–34]
ARDS Etiology	
*Pneumonia, n. (%)*	15 (83)
*Autoimmune disease, n. (%)*	1 (6)
*Trauma, n. (%)*	1 (6)
*Other, n, (%)*	1 (6)
Pneumonia Etiology	
*Bacterial, n. (%)*	11 (73)
*Viral, n. (%)*	3 (20)
*Unknown, n. (%)*	1 (7)
SOFA score	10 [7–12]
PaO2/FiO2 ratio before ECMO start	65.5 [55.7−86.5]
History of chronic kidney disease, *n*. (%)	3 (17)
Risk factors for chronic kidney disease	
*Hypertension, n. (%)*	6 (33)
*Diabetes, n. (%)*	4 (22)
ECMO days before randomization	0 [0−4]
Patients on CRRT before ECMO, *n*. (%)	6 (29)
Days from hospital admission to ECMO	4.5 [2–12]
ECMO duration, days	11 [7−23.5]
Patients weaned from ECMO, *n*. (%)	12 (67)
Patients discharged alive from ICU, *n*. (%)	10 (56)
**Liver function**	
AST, U/L	78 [44−175]
ALT, U/L	37 [21−66]
Bilirubin, mg/dl	1.3 [0.6−2.5]
ALP, U/L	102 [49−193]
GGT, U/L	292 [31−395]
LDH, U/L	488 [366−669]
**Hemodynamics**	
HR, bpm	95 [83−109]
SAP, mmHg	105 [94−121]
MAP, mmHg	72 [66−81]
DAP, mmHg	55 [51−63]
CVP, mmHg	8 [6–12]
sPAP, mmHg	38 [33−47]
mPAP, mmHg	28 [25−38]
dPAP, mmHg	19 [15–22]
WP, mmHg	14 [10–16]
CO, L/min	6.3 [5.0−9.3]
**Vasopressors dosage**	
Noradrenaline, mcg/Kg/min	0.14 [0.1−0.34]
Dopamine, mcg/Kg/min	0 [0−0]
Dobutamine, mcg/Kg/min	4.4 [2.2−6.7]
**Blood cell count**	
Haemoglobin, mg/dL	9.9 [8.9−10.5]
Haematocrit, %	29.7 [26.6−31.7]
Platelets, x 10^9^/L	132.5 [71.7−202.7]
**Coagulation**	
UFH, UI/die	19,200 [12,000−24,000]
aPTTr	1.34 [1.26−1.67]
INR	1.12 [0.98−1.40]
Fibrinogen	561 [387−856]
Dimers	1551 [1113−3368]
ATIII	91 [67−135]
Haptoglobin, mg/dL	234 [139−346]
Activated clotting time	178 [165−184]

Data are presented as median [25th–75th percentile] or number (percentage). ARDS, acute respiratory distress syndrome; SOFA, Sequential Organ Failure Assessment; ECMO, extracorporeal membrane oxygenation; CRRT, continuous renal replacement therapy; AST, aspartate aminotransferase; ALT, alanine aminotransferase; ALP, alkaline phosphatase; GGT, gamma-glutamyl transferase; LDH, lactate dehydrogenase; HR, heart rate; SAP, systolic arterial pressure; MAP, mean arterial pressure; DAP, diastolic arterial pressure; CVP, central venous pressure; sPAP, systolic pulmonary artery pressure; mPAP, mean pulmonary artery pressure; dPAP, diastolic pulmonary artery pressure; WP, pulmonary capillary wedge pressure; CO, cardiac output; UFH, unfractionated heparin; aPTTr, activated partial thromboplastin time ratio; INR, international normalized ratio; ATIII, antithrombin III; ACT, activated clotting time.

UFH infusion rates were comparable between anticoagulation regimens, with median doses of 22,080 [15,360–29,040] IU/day during RCA plus UFH and 22,320 [15,360–28,800] IU/day during UFH alone (*p* = 0.610). Systemic anticoagulation intensity did not differ between groups, as reflected by similar aPTT ratios (1.28 [1.12–1.45] vs. 1.29 [1.19–1.43]; *p* = 0.530), anti–Xa activity (0.25 [0.18–0.34] vs. 0.26 [0.18–0.33] IU/mL; *p* = 0.095), and ACT values (168 [158–176] vs. 166 [155–177] seconds; *p* = 0.872).

Circuit pressure data were available for 1941 time points, and their temporal trends according to anticoagulation strategy are shown in Fig. S2 (Supplementary material), with measurements stratified by the occurrence of circuit clotting. Overall, pressure drop across the RRT filter (indexed on the CRRT blood flow) was lower in the RCA + UFH group compared to UFH alone: 20 [8,[17], [18], [19], [20], [21], [22], [23], [24]] vs. 25 [20–30] mmHg/100 mL/min, *p* < 0.001, respectively.

**Fig. 2:**
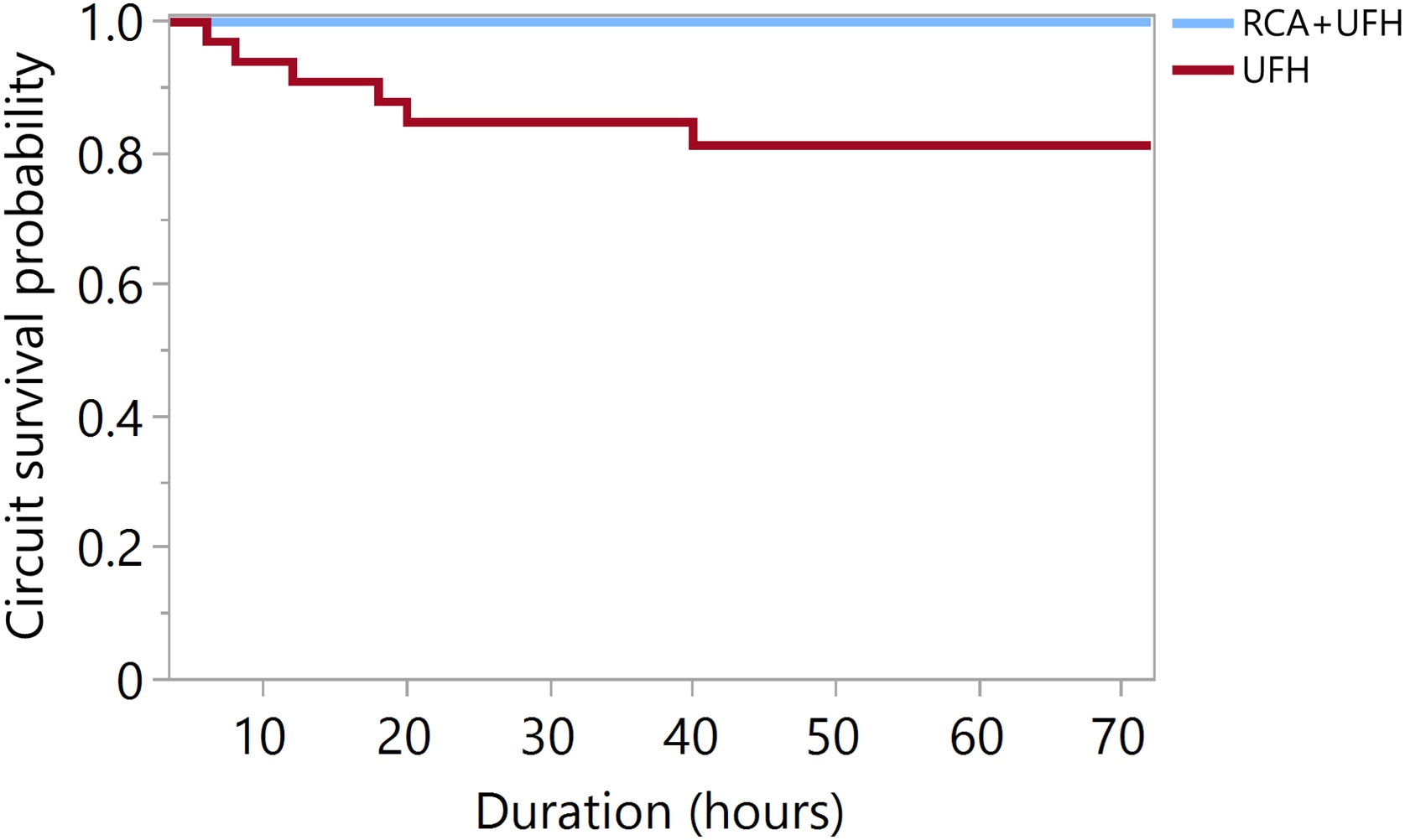
CRRT circuit survival according to anticoagulation strategy. Kaplan–Meier curves showing continuous renal replacement therapy (CRRT) circuit survival stratified by anticoagulation regimen. Circuits anticoagulated with RCA + UFH showed significantly longer survival compared with circuits anticoagulated with UFH alone (Cox proportional hazards model, *p* = 0.029). Circuit clotting was defined as the event of interest; circuits discontinued for reasons other than clotting (including ECMO discontinuation, CRRT discontinuation, or patient death) were right-censored. Abbreviations: RCA: regional citrate anticoagulation; UFH: unfractionated heparin.

No clotting events occurred in the RCA + UFH group, whereas 6 episodes of circuit clotting were observed with UFH alone (0% vs 18%, *p* < 0.001). Cox proportional hazards analysis showed significantly longer circuit survival in the RCA + UFH group compared to UFH alone (*p* = 0.029). Kaplan–Meier curves illustrating circuit survival over time are provided in Fig. 2. Details regarding the causes of circuit interruption other than clotting in the two groups are provided in the Supplementary material (Table S1). Detailed pressure parameters at the time of circuit clotting are reported in Table S2.

**Fig. 3:**
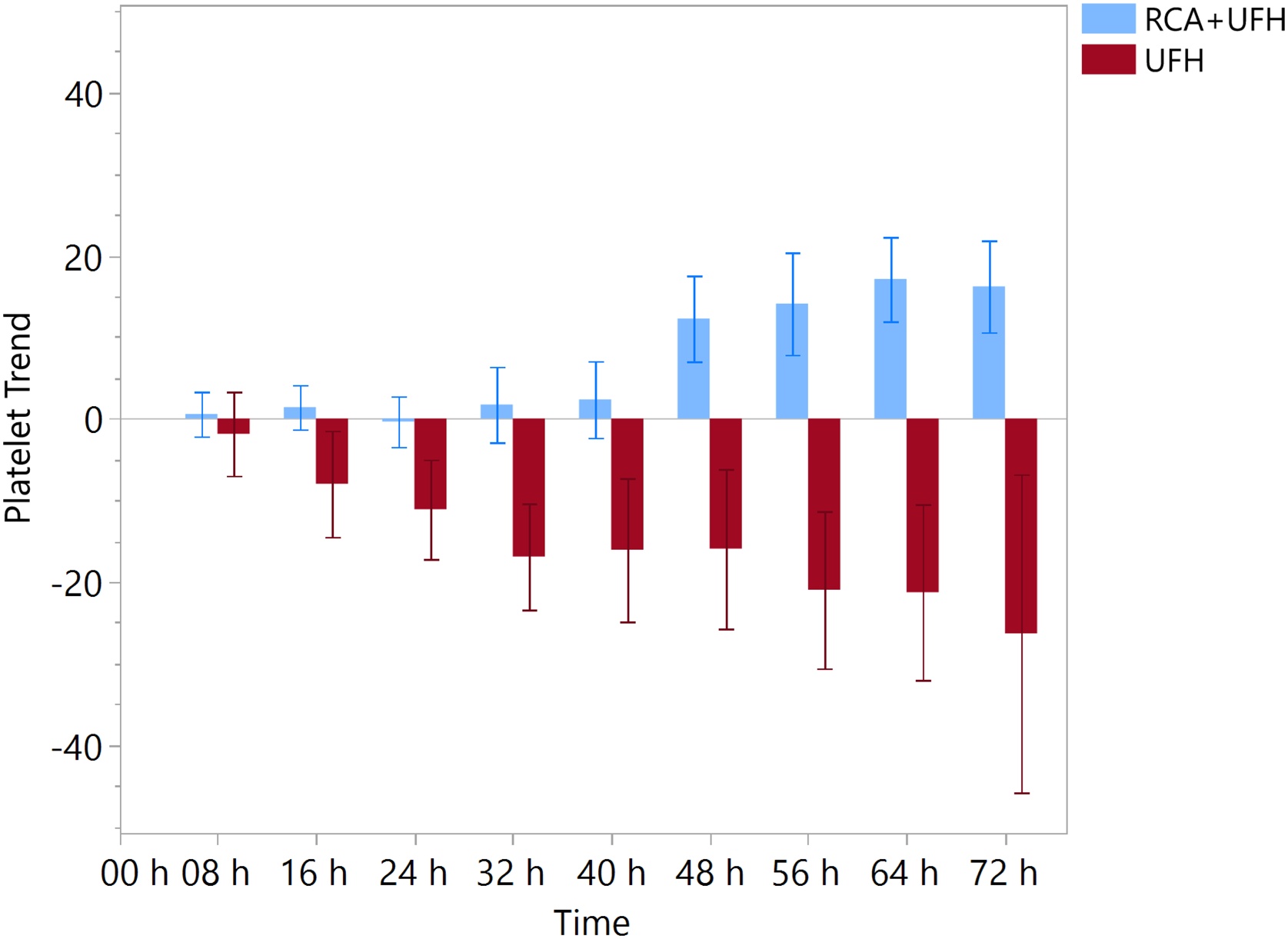
Changes in platelet count over time according to the CRRT anticoagulation regimen. Values are expressed as mean change from circuit baseline (start of each CRRT circuit), with error bars representing standard error. Blue bars indicate circuits anticoagulated with RCA + UFH, and red bars indicate circuits anticoagulated with UFH alone. Time points are shown at 8-h intervals up to 72 h. Abbreviations: RCA: regional citrate anticoagulation; UFH: unfractionated heparin.

Changes in platelet count over time according to the CRRT anticoagulation regimen are shown in Fig. 3. Platelet counts declined during CRRT circuits anticoagulated with UFH alone, whereas they increased during circuits anticoagulated with RCA + UFH. When expressed as change from circuit baseline, platelet counts showed a significantly more favorable overall trend with RCA + UFH compared with UFH alone (effect estimate: +13 × 10³/μL 95% CI (8–19) for RCA + UFH vs UFH, *p* < 0.001). A period effect was also recorded, with earlier circuits associated with a lower platelet count (effect estimate = 4.5–95% CI 2.5–6.5, *p* < 0.001), whereas the randomization sequence was not associated with platelet change (*p* = 0.229).

No significant differences were observed between anticoagulation strategies in fibrinogen trend over time (effect estimate: +8 mg/dL 95% CI (−36 to 19) for RCA + UFH vs UFH, *p* = 0.538), whereas dimers increased significantly in the UFH group (effect estimate: −782 μg/L 95% CI (−1525 to −39) for RCA + UFH vs UFH, *p* = 0.039). Platelet transfusion requirements were comparable between groups (0 [0–1] platelet pool in both groups; *p* = 0.157), and haptoglobin levels did not differ significantly (104 [40–263] vs 122 [6–205] mg/dL, *p* = 0.444).

**Fig. 4:**
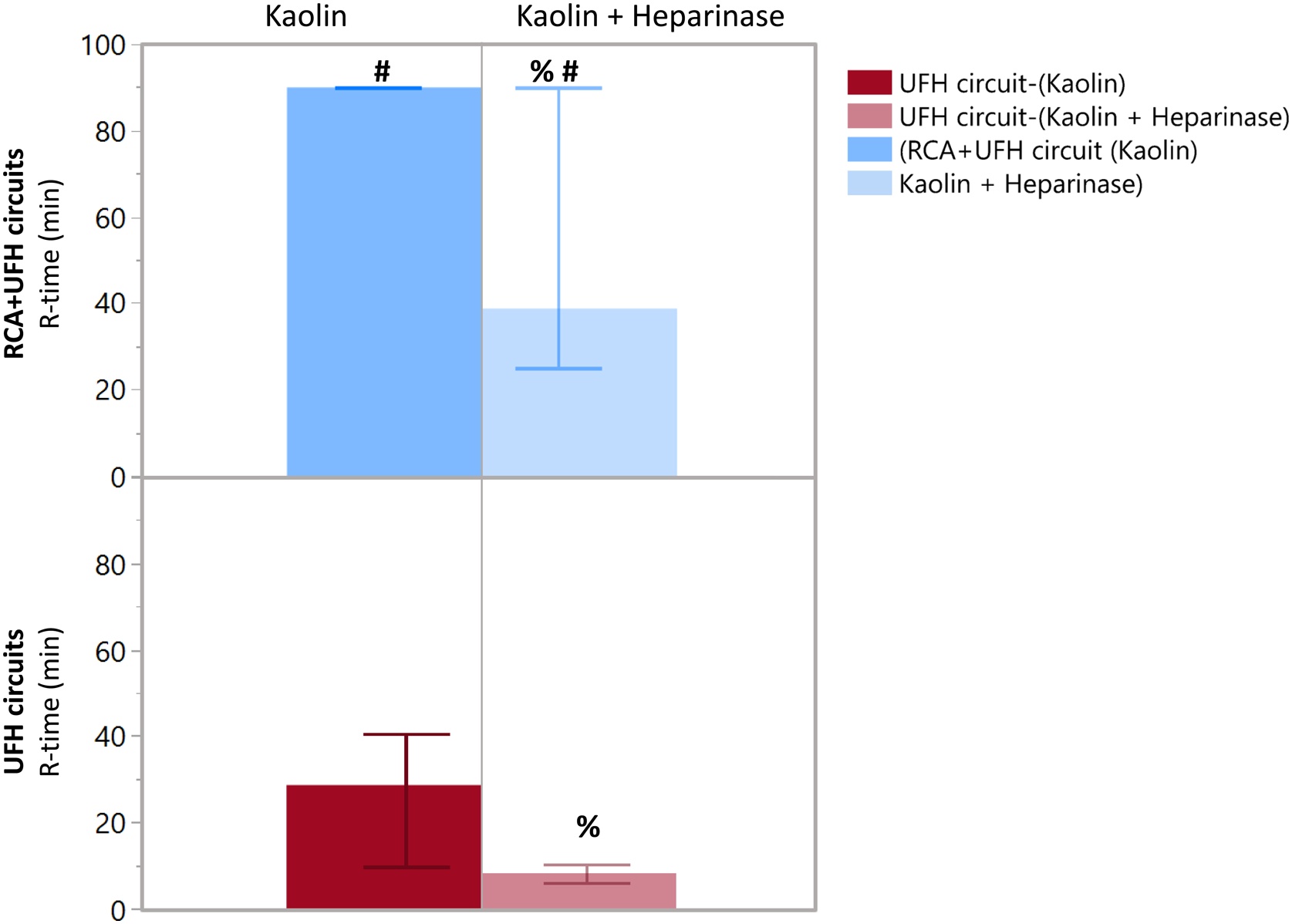
Thromboelastography R-time according to anticoagulation strategy and reagent. Thromboelastographic tests were performed on blood samples drawn directly from CRRT circuits anticoagulated with UFH alone or with RCA + UFH. Analyses were conducted using kaolin activation and kaolin plus heparinase to distinguish the regional anticoagulant effect of citrate from the effect of systemic heparin. Data are presented as median and interquartile range. % *p* < 0.05 vs kaolin; # *p* < 0.05 vs UFH circuits. Abbreviations: CRRT = continuous renal replacement therapy; R-time = reaction time; RCA = regional citrate anticoagulation; UFH = unfractionated heparin.

Fig. 4 illustrates the anticoagulant effects of RCA + UFH compared to UFH alone on CRRT circuits. TEG analysis revealed a significantly prolonged R-time in circuits anticoagulated with RCA + UFH compared to those with UFH alone (median R-time: 90 [90–90] vs. 30 [15–48] minutes; *p* < 0.001). When heparin was neutralized with heparinase, the R-time remained significantly longer in the RCA + UFH circuits compared to UFH alone (36 [26–90] vs. 8 [[6], [7], [8], [9], [10]] minutes; *p* < 0.001).

No metabolic complications related to RCA were observed. Specifically, no episodes of severe hypernatremia, metabolic alkalosis, or citrate accumulation occurred. Episodes of moderate hypernatremia were infrequent and occurred in 6 of 253 blood samples (5.3%) during RCA + UFH and in 4 of 225 samples (4.7%) during UFH alone (*p* = 0.205). Although serum sodium concentrations remained within the normal range in both groups, values were modestly but significantly higher during RCA + UFH compared with UFH alone (141 [139–143] vs. 137 [135–139] mmol/L; *p* < 0.001). When RCA was used, the total-to-ionized calcium ratio consistently remained below the safety threshold of 2.5, with a median value of 1.82 [1.76–1.92] across all measurements.

Small but statistically significant differences in selected laboratory parameters were observed. Total serum calcium levels were slightly higher in the RCA + UFH group (8.8 [8.6–9.2] vs. 8.4 [8.2–8.7] mg/dL; *p* < 0.001). Similarly, acid–base parameters showed a mild alkalinizing effect with RCA + UFH, reflected by higher pH (7.41 [7.38–7.45] vs. 7.39 [7.37–7.42]; *p* < 0.001), bicarbonate (30.1 [27.1–32.0] vs. 27.7 [26.0–29.6] mmol/L; *p* < 0.001), and standard base excess (5.3 [2.3–8.2] vs. 2.6 mmol/L [0.9–4.5]; *p* < 0.001). All values remained within clinically acceptable ranges.

Other biochemical parameters, including complete blood count and liver and renal function tests, were largely comparable between anticoagulation strategies. ECMO configuration, respiratory settings, respiratory mechanics, and hemodynamic parameters were also similar. All additional results are reported in the Supplementary Results (Table S3).

## Discussion

In this randomized crossover pilot study of patients supported with VV ECMO, the addition of regional citrate anticoagulation to systemic unfractionated heparin was associated with a reduction in CRRT circuit clotting, supporting a combined anticoagulation strategy in this high-risk population.

Citrate is currently recommended as the preferred anticoagulant for CRRT [7]. However, in many ECMO centres, when systemic heparinization is already ongoing, no additional anticoagulation is generally used for CRRT [21]. Citrate provides supplemental regional anticoagulation by chelating ionized calcium (Ca²^+^), an essential cofactor in several steps of the coagulation cascade, thereby inhibiting both coagulation factor activation and platelet aggregation [22]. Its effect is limited to the extracorporeal circuit, as citrate is rapidly metabolized – mainly in the liver, muscle, and kidneys – into bicarbonate, leading to dissociation of the calcium-citrate complex and partial restoration of Ca^2+^ in the systemic circulation, with additional calcium provided through continuous infusion via the CRRT post-filter line [22,23].

In line with our previous retrospective study [20], our results highlight the potential benefits of a combined anticoagulation strategy in this critically ill population.

Regional citrate anticoagulation reduced CRRT circuit clotting despite comparable levels of systemic anticoagulation between study phases. Notably, UFH dosage, aPTT ratio, anti–factor Xa activity, and ACT values did not differ between anticoagulation regimens, indicating that the observed benefit was not driven by intensified systemic anticoagulation. This effect was accompanied by reduced circuit-related platelet consumption, a finding particularly relevant in ECMO patients, in whom thrombocytopenia is common [14] and is a well-recognized risk factor for bleeding [15]. During RCA + UFH, platelet count trajectories were more favorable over the course of CRRT circuits compared with UFH alone. This finding should not be interpreted as a direct platelet-increasing effect of citrate, but rather as a consequence of reduced platelet activation and consumption within the extracorporeal circuit. In the crossover design of the present study, platelet counts typically declined during UFH-only circuits and subsequently stabilized or increased following the introduction of RCA, consistent with improved circuit biocompatibility.

To our knowledge, no studies have directly compared the addition of RCA to systemic heparinization in critically ill patients undergoing VV ECMO. A recent meta-analysis by Zou et al. [5], including 37 RCTs on critically ill patients not receiving ECMO, showed that RCA alone was more effective than UFH in prolonging filter lifespan and reducing bleeding risk. These findings may lead us to hypothesize that adopting a regional anticoagulation strategy in patients already receiving systemic UFH could help counteract the procoagulant impact of the CRRT circuit in ECMO patients. Notably, the circuit lifespan observed in our study is similar to that reported in the recent E-CRRT randomized trial [19], helping to contextualize our findings within contemporary ECMO practice.

In our study, this concept is further supported by TEG analysis, which revealed a significantly prolonged R-time in samples collected from circuits anticoagulated with RCA compared to those treated with UFH alone. This augmented local anticoagulant effect, attributable to the synergistic action of citrate and heparin, likely accounts for the complete absence of clotting events observed in the RCA + UFH group. Importantly, systemic coagulation profiles were similar between the two groups, confirming that the anticoagulant effect of RCA is confined to the extracorporeal circuit.

Beyond its beneficial effect on circuit patency, RCA proved safe in our cohort, with no significant rise in citrate-related metabolic complications—despite theoretical concerns about calcium shifts and impaired citrate metabolism [10,22]. While small but statistically significant differences were observed in total calcium and acid-base parameters, these changes remained well within normal physiological ranges and were consistent with the expected metabolic effects of citrate [24]. However, this favorable safety profile observed in our cohort may partly reflect a selection bias, as our strict ECMO eligibility criteria exclude patients with severe hepatic failure or advanced end-organ dysfunction, who are most at risk for citrate accumulation and toxicity.

Taken together, these findings not only support the biological rationale for combining RCA with systemic UFH but also highlight its clinical relevance. Frequent clotting of the CRRT circuit disrupts continuous therapy, contributes to increased blood loss and blood components needs, and ultimately leads to higher healthcare costs and resource consumption [5,25]. The combined anticoagulation approach may represent a feasible and potentially more effective strategy to optimize CRRT management in high-risk VV ECMO patients.

Some limitations should be acknowledged. First, the study was prematurely terminated due to lack of funding for study insurance, resulting in a smaller-than-planned sample size. This represents an important limitation that may affect the robustness and generalizability of the findings and should be considered when interpreting the results. Although a statistically significant difference in circuit clotting was observed between the two anticoagulation strategies, the limited number of patients and the relatively small number of events inherently imply that the risk of type I error cannot be excluded. Accordingly, the observed findings should be interpreted with caution and considered hypothesis-generating rather than definitive.

Second, its single-centre design and relatively small sample size may further limit the generalizability of the results. While the randomized crossover design reduces interpatient variability and enhances internal validity, multicentre studies are needed to confirm these findings across different clinical settings.

Third, the definition of circuit clotting is not uniformly standardized, particularly in the context of integrated ECMO–CRRT systems. In our study, clotting was defined based on clinically relevant circuit dysfunction rather than a single pressure threshold. While this pragmatic approach reflects real-world practice, it may limit direct comparability with studies adopting predefined transmembrane pressure cut-offs. Notably, pressure thresholds derived from standalone CRRT systems may not be directly applicable to ECMO-integrated configurations, where circuit pressures are influenced by extracorporeal flow dynamics.

Fourth, although the statistical analysis plan was prespecified, it was not publicly available prior to study completion; however, key methodological elements, including primary endpoints and main analytical approaches, were predefined and available in the ClinicalTrials.gov registry.

Finally, we did not assess specific clinical outcomes, such as bleeding complications or long-term patient-centered outcomes, which should be explored in future studies to better define the clinical impact of these findings.

## Conclusions

The addition of RCA to systemic UFH was associated with improved CRRT lifespan and reduced circuit clotting in patients undergoing VV ECMO. This combined strategy appears both feasible and safe, with no evident increase in complications. While these findings are promising, they should be interpreted with caution given the pilot nature of the study and require confirmation through larger multicentre trials before broader clinical application.

## Consent for publication

Not applicable.

## Ethical approval and consent to participate

The study was approved by the institutional ethics committee (October 2021; ref. 3732). Delayed informed consent was obtained from all patients once they regained consciousness, in accordance with ethical requirements. The study design and all endpoints were predefined and prospectively registered on ClinicalTrials.gov (NCT05148026).

## Funding

The study received no external funding. Study insurance was covered by institutional funds from the University of Milano-Bicocca.

## Availability of supporting data

The datasets generated and/or analyzed during the current study are available from the corresponding author (M.G.) upon reasonable request.

## CRediT authorship contribution statement

**Marco Giani:** Conceptualization, Methodology, Formal analysis, Investigation, Data curation, Writing - original draft, Supervision, Project administration. **Marta Frazzei:** Formal analysis, Investigation, Data curation, Writing - original draft. **Roberto Rona:** Conceptualization, Writing - review & editing. **Thomas Langer:** Writing - review & editing. **Matteo Pozzi:** Writing - review & editing. **Giuseppe Foti:** Writing - review & editing. **Emanuele Rezoagli:** Methodology, Formal analysis, Writing - review & editing.

## Declaration of competing interest

The authors declare no conflicts of interest related to this work.
