# Supplementary material for: Regional citrate anticoagulation for renal replacement therapy during venovenous ECMO: A randomized crossover pilot study
Source: Ann Intensive Care. 2026 Apr 27;16:100072. doi: 10.1016/j.aicoj.2026.100072 (PMC13137002; doi:10.1016/j.aicoj.2026.100072)
Supplement: Supplementary file 1 [file mmc1.pdf]

Supplementary Material

Regional Citrate Anticoagulation for Renal Replacement Therapy During Venovenous ECMO: A Randomized Crossover Pilot Study

Marco Giani MD[1,2], Marta Frazzei MD[1,2], Roberto Rona[2], Thomas Langer[1,3], Matteo Pozzi MD[1,2], Giuseppe Foti MD[1,2] Emanuele Rezoagli MD, PhD[1,2], and the CRRT ECMO Study Group

1. Department of Medicine and Surgery, University of Milano-Bicocca, Monza, Lombardy, Italy.
2. Emergency and Intensive Care, Fondazione IRCCS San Gerardo dei Tintori, Monza, Lombardy, Italy.
3. Emergency and Intensive Care Medicine, ASST Grande Ospedale Metropolitano Niguarda, Milano, Lombardy, Italy

SUPPPLEMENTARY METHODS

ECMO MANAGEMENT

Cannulation was performed percutaneously. Drainage and reinfusion were achieved using Maquet HLS cannulas (21–25 Fr; Getinge, Göteborg, Sweden) and Bio-Medicus cannulas (17–23 Fr; Medtronic, Minneapolis, MN), respectively.

The ECMO circuit consisted of a centrifugal pump, a polymethylpentene membrane oxygenator, and heparin-coated tubing (BIOLINE; Maquet GmbH, Rastatt, Germany). Either the Permanent Life Support (PLS) system or the CARDIOHELP system (Maquet, Rastatt, Germany) was used according to device availability.

ANTICOAGULATION PROTOCOL

Systemic anticoagulation for ECMO was initiated with an intravenous bolus of unfractionated heparin (UFH) (50–100 IU/kg) before cannulation. After ECMO connection, continuous heparin infusion was started and initially titrated to maintain an activated clotting time (ACT) between 170 and 200 seconds, assessed by point-of-care testing (Hemochron Jr. Signature+, ITC, Piscataway, NJ).

After an initial 12-hour stabilization period, anticoagulation was guided by activated partial thromboplastin time (aPTT) and anti-factor Xa activity (anti-Xa), targeting an aPTT ratio of approximately 1.5 or an anti-Xa level between 0.2 and 0.4 IU/mL. Antithrombin III (ATIII) activity was routinely

monitored, and supplementation was administered when levels fell below the normal range (80–100%) to optimize heparin efficacy.

Complete blood count, ACT, and standard coagulation parameters—including aPTT, prothrombin time (PT), fibrinogen, and D-dimers—were measured three times daily.

CRRT MANAGEMENT

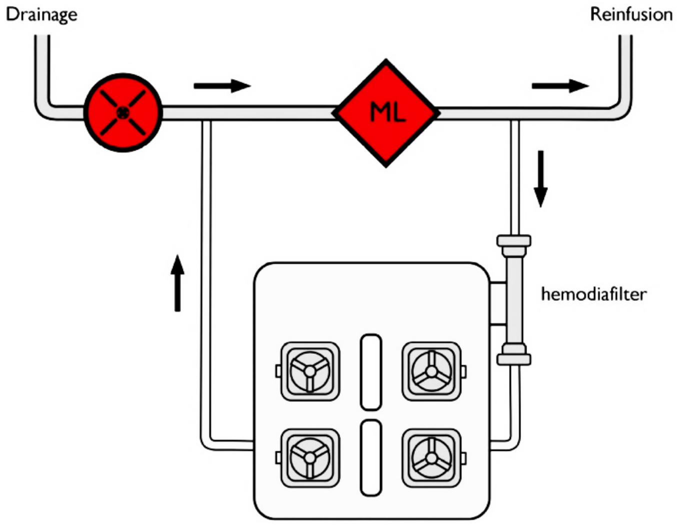

Figure S1. Schematic of the connection of the CRRT device to ECMO circuit. Modified from "Continuous Renal Replacement Therapy in Venovenous Extracorporeal Membrane Oxygenation: A Retrospective Study on Regional Citrate Anticoagulation. ASAIO Journal 66(3): p 332-338, March 2020". List of abbreviation: ML: membrane lung.

CRRT was connected to the ECMO circuit as illustrated in **Figure S1**. When systemic UFH was used as the sole anticoagulant, CRRT was performed in continuous venovenous hemodialysis (CVVHD) mode using a polysulfone membrane (Ultraflux AV600; surface area 1.4 m^2, priming volume 100 mL, molecular cutoff 30 kDa). Blood flow was set at 200ml/min, while dialysate flow was adjusted according to clinical needs.

During regional citrate anticoagulation, systemic heparinization remained unchanged. CRRT was performed in CVVHD mode using the Ci–Ca MultiFiltrate configuration with an Ultraflux AV1000 membrane (surface area 1.8 m^2, priming volume 130 mL, molecular cutoff 30 kDa). Blood flow was maintained between 100 and 120 mL/min, and a calcium-free dialysate was used.

A 4% trisodium citrate solution (Fresenius; citrate concentration 136 mmol/L) was infused into the prefilter line to achieve a target postfilter ionized calcium concentration of 0.25–0.34 mmol/L. Citrate infusion rates typically ranged from 130 to 260 mL/h, corresponding to prefilter citrate concentrations of approximately 3–5 mmol/L.

To compensate for calcium loss, a calcium chloride solution (100 mmol/L) was infused into the return line at 20–50 mL/h, titrated to maintain systemic ionized calcium between 1.12 and 1.20 mmol/L. Postfilter and systemic ionized calcium levels were monitored at least every 6 hours and after any change in CRRT settings.

SUPPLEMENTARY RESULTS

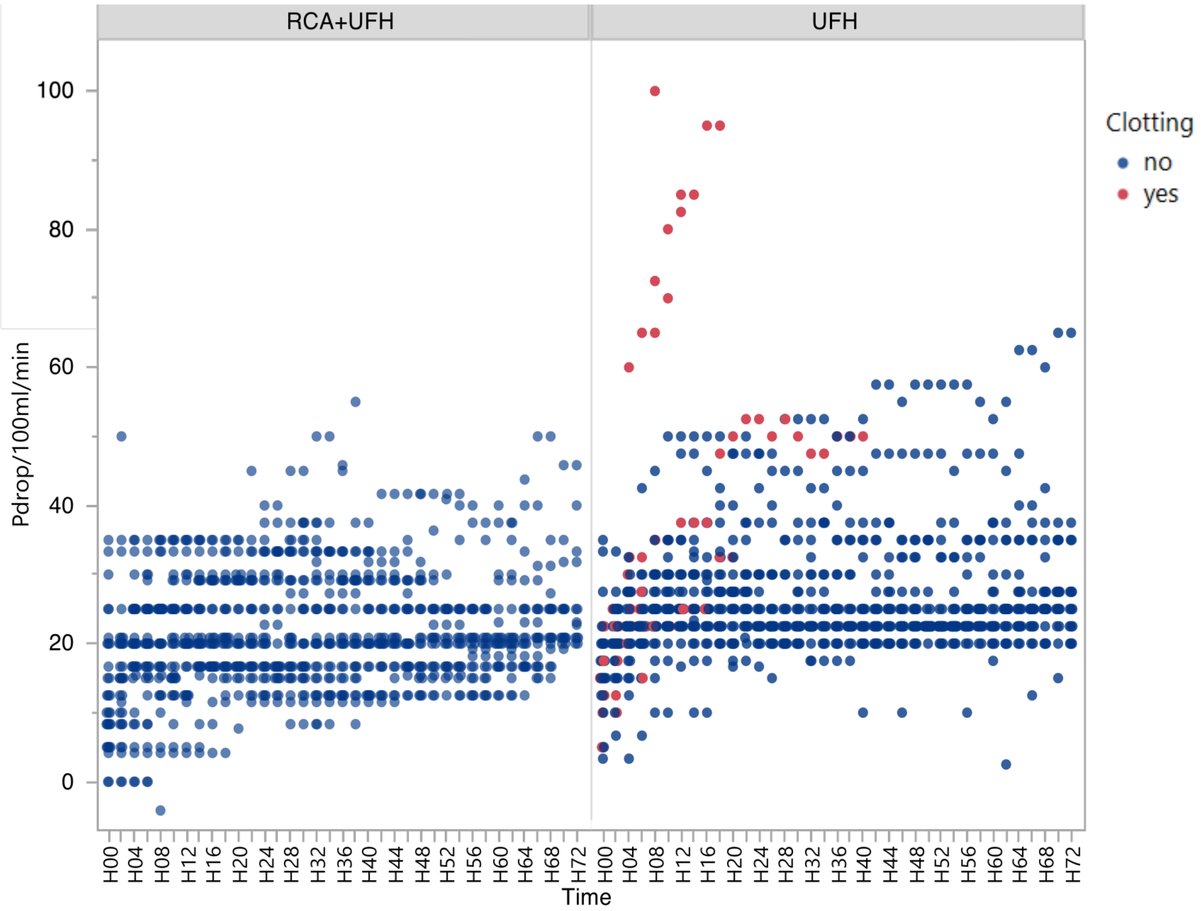

Figure S2. Temporal trends of pressure drop across the CRRT filter indexed to blood flow (mmHg/100 mL/min) according to anticoagulation strategy. Individual measurements are shown for circuits anticoagulated with regional citrate anticoagulation plus unfractionated heparin (RCA + UFH) and UFH alone**.** Each point represents a single time point and is stratified according to the occurrence of circuit clotting (**red dots**) or absence of clotting (**blue dots**).

Table S1

Causes of CRRT circuit interruption		
	**RCA+UFH**	**UFH**
Clotting, n. (%)	0 (0.0)	6 (18.2)
Elective replacement, n. (%)	20 (60.6)	19 (57.6)
ECMO discontinuation, n. (%)	5 (15.2)	7 (21.2)
ECMO circuit replacement, n. (%)	4 (12.1)	1 (3.0)
Patient exitus, n. (%)	2 (6.1)	0 (0.0)
Procedures outside the ICU, n. (%)	1 (3.0)	0 (0.0)
CRRT machine malfunction, n. (%)	1 (3.0)	0 (0.0)

Table S1. Causes of CRRT discontinuation. Elective replacement was performed at 72 hours, as per manufacturer recommendations. Values are reported as absolute number (percentage). Abbreviations: ECMO, extracorporeal membrane oxygenation; ICU, intensive care unit; CRRT, continuous renal replacement therapy.

Table S2

Patient	Circuit	Regimen	Time (h)	Drainage pressure		Prefilter pressure		Postfilter pressure		ΔP pressure		Visible Clot
				Start	End	Start	End	Start	End	Start	End	
**228**	1	UFH	20	10	**15**	210	**280**	175	**180**	35	**100**	Yes
**251**	4	UFH	18	80	**95**	180	**260**	175	**165**	10	**95**	Yes
**251**	5	UFH	12	55	**105**	250	**430**	230	**265**	20	**165**	No
**259**	4	UFH	40	115	**95**	305	**315**	260	**215**	45	**100**	Yes
**269**	1	UFH	8	-55	**-60**	125	**475**	100	**275**	25	**200**	No
**269**	3	UFH	6	-20	**55**	190	**550**	160	**520**	30	**30**	Yes

Table S2: Characteristics of CRRT circuits classified as clotted (UFH group). Detailed characteristics of CRRT circuits classified as clotted in the UFH group, including circuit duration, operational settings, and pressure parameters at the time of clotting. List of abbreviations: UFH, unfractionated heparin; Qb, blood flow; Qd, dialysate flow; ΔP, pressure drop (difference between prefilter and postfilter pressure).

Table S3

	RCA+UFH	UFH	*Difference (RCA+UFH vs UFH), estimate (95% CI)*
**Hematological and biochemical parameters**			
WBC (10^9/L)	13 [9.1-19.2]	12.7 [7.9-17.6]	0.09 (−0.96 to 1.15)
Hb (g/dL)	9.4 [8.9-9.9]	9.3 [8.9-10.0] *	−0.19 (−0.30 to −0.07)
Ht (%)	28.4 [26.7-29.9]	28.1 [26.7-30.1]	−0.56 (−0.92 to −0.19)
Creatinine (mg/dL)	1.1 [0.8-1.4]	1.1 [0.8-1.7]	0.02 (−0.10 to 0.14)
Urea (mg/dL)	74 [51.2-105.0]	74 [52.0-97.5]	6.00 (−3.90 to 15.91)
Serum calcium (mg/dL)	8.8 [8.6-9.2]	8.4 [8.2-8.7] *	0.33 (0.19 to 0.48)
ALT (U/L)	39.0 [23.0-60.0]	40 [22.0-70.5]	−63.55 (−177.11 to 50.00)
AST (U/L)	62.5 [43.3-111]	85 [40.0-166.5]	−350.47 (−744.56 to 43.62)
Bilirubin (mg/dL)	1.4 [0.6-2.9]	1.6 [0.6-2.9]	−0.014 (−0.29 to 0.27)
ALP (U/L)	211 [146.5-311]	215.0 [109.7-271.7]	11.29 (−28.00 to 50.59)
GGT (U/L)	349 [167.3-551.7]	318 [145-576]	11.26 (−73.98 to 96.51)
LDH (U/L)	454 [369-496]	470.0 [396.0-579.0]	−206.04 (−454.21 to 42.13)
CRP (mg/dL)	11.3 [6.2-18.1]	13.7 [6.2-28.7] *	−7.80 (−13.77 to −1.83)
PCT (ng/mL)	2.4 [1.3-11.1]	3.7 [1.7-16.7]	−11.54 (−26.10 to 3.02)
**Anticoagulation**			
UFH daily dose (IU)	22,080 [15,360–29,040]	22,320 [15,360–28,800]	232.50 (−663.04 to 1128.03)
aPTT ratio	1.28 [1.12–1.45]	1.29 [1.19–1.43]	232.50 (−663.04 to 1128.03)
antiXa activity (IU/mL)	0.25 [0.18–0.34]	0.26 [0.18–0.33]	−0.028 (−0.061 to 0.005)
Activated Clotting Time (sec)	168 [158–176]	166 [155–177]	−0.21 (−2.81 to 2.38)
**Arterial blood gas analysis**			
pO2 (mmHg)	79.6 [69.3-92.0]	82.0 [72.6-96.0]	−2.34 (−4.79 to 0.11)
pCO2 (mmHg)	47.0 [43.0-53.3]	46.3 [42.0-50.3]	0.52 (−0.37 to 1.41)
pH	7.41 [7.38-7.45]	7.39 [7.37-7.42] *	0.02 (0.01 to 0.03)
HCO3- (mmol/L)	30.0 [27.1-32.7]	27.8 [26.1-29.6] *	1.58 (1.03 to 2.14)
Base Excess (mmol/L)	5.2 [2.4-8.1]	2.8 [1.3-4.6] *	1.67 (1.04 to 2.29)
Lactate (mmol/L)	1.7 [1.3-2.2]	1.5 [1.2-2.1]	−0.06 (−0.25 to 0.14)
Sodium (mmol/L)	141 [139-143]	137 [135-139] *	3.36 (2.80 to 3.93)
Potassium (mmol/L)	4.4 [4.2-4.8]	4.6 [4.4-4.9] *	−0.12 (−0.19 to −0.06)
Chloride (mmol/L)	106 [104-107]	104 [103-105] *	1.42 (1.08 to 1.77)
**Hemodynamic parameters**			

HR (bpm)	100 [86-106]	95 [85-102]	1.11 (−0.88 to 3.10)
SAP (mmHg)	113 [102-126]	112 [98-125]	2.01 (−0.97 to 5.00)
MAP (mmHg)	75 [67-84]	74 [66-82]	1.12 (−0.91 to 3.15)
DAP (mmHg)	56 [50-63]	56 [50-63]	0.11 (−1.51 to 1.73)
CVP (mmHg)	9 [6-12]	8 [5-11]	0.11 (−1.51 to 1.73)
sPAP (mmHg)	41 [32-50]	41 [33-49]	−0.56 (−1.79 to 0.68)
mPAP (mmHg)	30 [22-35]	27 [22-35]	0.15 (−0.71 to 1.02)
dPAP (mmHg)	19 [14-24]	18 [13-23]	0.21 (−0.56 to 0.97)
WP (mmHg)	13 [11-15]	12 [10-14] *	0.67 (0.03 to 1.31)
CO (L/min)	7.4 [5.8-9.5]	6.6 [5.6-8.6] *	0.53 (0.31 to 0.76)
**Vasopressors**			
Noradrenaline (mcg/kg/min)	0.11 [0.06-0.14]	0.11 [0.06-0.17] *	−0.033 (−0.053 to −0.014)
Dobutamine (mcg/kg/min)	4.55 [2.18-7.56]	3.96 [3.22-7.56]	−0.29 (−0.82 to 0.23)
**ECMO settings**			
Blood flow (L/min)	3.4 [3.1-3.8]	3.3 [3.1-3.7]	0.11 (−0.01 to 0.23)
Sweep Gas (L/min)	4.5 [3.5-5.5]	4.5 [3.0-5.5]	−0.34 (−0.69 to 0.02)
FiO_2 ML (%)	65 [50-95]	60 [50-80]	0.93 (−4.43 to 6.29)
P drain (mmHg)	-42 [-54 to -30]	-40 [-53 to –27.5]	−0.81 (−3.82 to 2.21)
P pre (mmHg)	118 [100-142]	114 [100 -136] *	7.16 (0.23 to 14.08)
P post (mmHg)	100 [83-123]	93 [78-116] *	6.90 (0.73 to 13.07)

Table S3. Biochemical, arterial blood gas, hemodynamic, vasopressor, and ECMO parameters measured during continuous renal replacement therapy (CRRT) circuits anticoagulated with combined regional citrate anticoagulation plus unfractionated heparin (RCA + UFH) or with UFH alone.

Values are reported as median [interquartile range]. Difference refer to measurements obtained during each anticoagulation phase within the randomized crossover design. Abbreviations: WBC, white blood cell count; Hb, hemoglobin; Hct, hematocrit; ALP, alkaline phosphatase; GGT, gamma-glutamyl transferase; CRP, C-reactive protein; PCT, procalcitonin; HR, heart rate; SAP, systolic arterial pressure; MAP, mean arterial pressure; DAP, diastolic arterial pressure; CVP, central venous pressure; sPAP, systolic pulmonary arterial pressure; mPAP, mean pulmonary arterial pressure; dPAP, diastolic pulmonary arterial pressure; WP, pulmonary capillary wedge pressure; CO, cardiac output; ML, membrane lung; P drain, drainage pressure (pre-pump pressure); P pre, pre-ML reinfusion pressure; P post, post-ML reinfusion pressure.
